# Fertility Awareness-Based Methods for Family Planning: A Systematic Review

**DOI:** 10.7759/cureus.86233

**Published:** 2025-06-17

**Authors:** Rasha A Bassas, Mohammad Saleh Alharbi, Shatha S Al Harbi

**Affiliations:** 1 Family and Community Medicine, Security Forces Hospital, Riyadh, SAU; 2 General Surgery, Riyadh Third Health Cluster, Riyadh, SAU; 3 Family Medicine, Princess Nourah Bint Abdul Rahman University, King Abdullah bin Abdulaziz University Hospital, Riyadh, SAU

**Keywords:** awareness, conception, contraception, family planning, fertility awareness-based methods

## Abstract

Fertility awareness-based methods (FABMs) have been used for a long time for family planning. This systematic review evaluates the efficacy and outcomes of various FABMs used for family planning. For this systematic review, a literature search was carried out in PubMed, Web of Science, CINAHL Ultimate, and Google Scholar. The inclusion criteria included women aged 18-49 undergoing FABMs for either contraception or to conceive. The search was limited from 2014 to 2024. The risk of bias was assessed using the Newcastle-Ottawa Scale (NOS) and the Revised Cochrane risk of bias tool for randomized trials (ROB2). A total of 16 studies, including 20,339 participants, were included. The age of the participants ranged from 18 to 47 years. Regarding study design, 11 were prospective, two were retrospective, two were randomized, and one was a longitudinal study. The average success rate of all FABMs was 69.5%. In five studies, the success rate was above 90%. Among factors that influenced the success rate were the timing of intercourse and adherence to method protocols. FABMs are effective tools for enhancing the success rate of family planning. However, FABMs when enhanced with digital technology are particularly effective for both contraception and conception. Adequate user education and consistent application are essential to optimize outcomes.

## Introduction and background

Family planning is crucial for empowering individuals and couples to make informed decisions about their reproductive health and ensure better maternal and child health outcomes. It helps reduce unintended pregnancies, lowers the risk of unsafe abortions, and supports economic stability by allowing families to plan for their future [1]. Fertility awareness-based methods (FABMs) of family planning comprise tracking numerous indications and symptoms of fertility throughout the menstrual cycle to determine the fertile window, or the days of the cycle when unprotected intercourse is most likely to result in pregnancy [2]. This contrasts with pharmacological or procedural interventions. FABMs track basal body temperature, cervical mucus changes, urinary hormone levels, or menstrual cycle patterns. These methods serve dual purposes as they can help avoid unintended pregnancies but also can help with conception [3].

FABM is essentially categorized into four types: (i) Calendar-based methods rely on cycle length and day counting, (ii) cervical mucus-based methods rely on observing and tracking vulvar discharge, (iii) symptom-thermal methods (STM) combine cervical mucus and basal body temperature observations, and (iv) symptom-hormonal methods combine mucus observation with technology to detect urinary hormonal metabolites associated with ovulation and fertility [4]. The utility of FABMs varies with region. For example, in the United States, FABMs are utilized by approximately 3% of contraceptive users [5]. However, a study conducted in Riyadh reported that 8.6% of women used the rhythm method (calendar method) [6]. FABMs also offer other advantages as well. They encourage partner involvement, improve communication, and enhance body literacy by tracking female biomarkers to determine fertility status. These methods positively influence relationships and help diagnose ovulation-related disorders, such as PCOS and endometriosis, which commonly affect reproductive health [7].

The most significant impediments to fertility awareness education in general practices are short consultations, time constraints for general practitioners, and a lack of patient educational resources and payment to support their delivery [8]. Despite their potential, FABMs are often underutilized and misunderstood in many regions, including both high-income and low-income countries [9]. In recent years, the prevalence of FABMs has risen, a trend that could be attributed to the rapid rise in population and adoption of digital apps for tracking the menstrual cycle [10]. Historically, FABMs were used as natural family planning methods where family planning was based on observing signs and symptoms. However, recently, the incorporation of digital technologies has led to the development of various FABM apps to track symptoms and cycles, which were previously done manually. This has led to an increase in the success rate [7].

Previously, systematic reviews have demonstrated the success of different FABMs in contraception and increasing conception rates [7,11]. However, these systematic reviews did not include the latest evidence on the topic. Therefore, this systematic review was conducted to comprehensively assess the effectiveness and outcomes of FABMs in family planning. The findings will help inform healthcare providers, policymakers, and individuals about the role of FABMs in modern family planning and guide future research in this important area of reproductive health.

## Review

Methods

This systematic review followed the guidelines of the Preferred Reporting Items for Systematic Reviews and Meta-Analyses (PRISMA) [12].

*Search Strategy* 

To identify relevant studies, PubMed, Web of Science, and CINAHL Ultimate were searched by using keywords related to fertility awareness-based methods for family planning. Furthermore, Google Scholar was also searched to further expand the list of potential studies. The keywords used in the search included ‘‘fertility awareness,’’ ‘‘natural family planning,’’ ‘‘rhythm method,’’ ‘‘cervical mucus method," "family planning," and "contraception". These keywords were combined by using AND and OR operators. Details of complete keyword combination are provided in Table 3 in the Appendices. 

Study Eligibility

The PICOS for the systematic review included population (P): women of reproductive age (18-49 years) who have utilized FABMs, interventions (I): any fertility awareness-based method for family planning, comparators (C): hormonal contraceptives (oral pills, patches, injections), long-acting reversible contraception (IUDs, implants), barrier methods (condoms, diaphragms), sterilization procedures, or no contraception use, outcomes (O): effectiveness, implementation success, and study design (S): randomized controlled trials (RCTs), prospective studies, and retrospective studies. To ensure the inclusion of the latest evidence, the systematic review focused on studies published and conducted between 2014 and 2024, investigating fertility awareness-based methods for family planning. The exclusion criteria included studies that did not investigate the efficacy of fertility awareness-based family planning methods and those that were not published in English language.

Study Selection

After retrieving results from the database search, the files were uploaded to a screening software, Rayyan [13]. In the first stage, duplicates were removed before beginning the screening process. Two independent reviewers were involved in the screening process. Both reviewers were blinded to each other’s decisions. During screening, records were first screened based on the title and abstract. After completing the initial screening, the blind was removed in Rayyan, and decisions were compared by other reviewers. In case a shared decision was not achieved, a third reviewer was involved. Finally, a full-length screening was performed. After making the final decision to include studies, the data was extracted from studies including demographic details and success rate.

Quality Assessment

The quality assessment was performed by two independent reviewers. For retrospective and prospective studies, the Newcastle-Ottawa Scale (NOS) tool was used [14]. For randomized studies, the Revised Cochrane risk of bias tool for randomized trials (ROB2) was used [15]. The NOS checklist has eight questions spread across three categories, which examine how well the study cohort represents the general population, whether confounding factors are controlled, and any biases in measuring outcomes.

Results

Included Studies

A total of 4193 studies were found during the initial database search. A total of 1341 studies were identified from PubMed, 2788 from Web of Science, 39 from CINAHL Ultimate, and 25 from Google Scholar. A total of 867 duplicates were removed before the screening process. During initial screening, 2762 studies were excluded based on the title and abstract of the records. Of the 557 studies further screened, only 16 studies met the inclusion criteria. 

Flow Diagram

**Figure 1:**
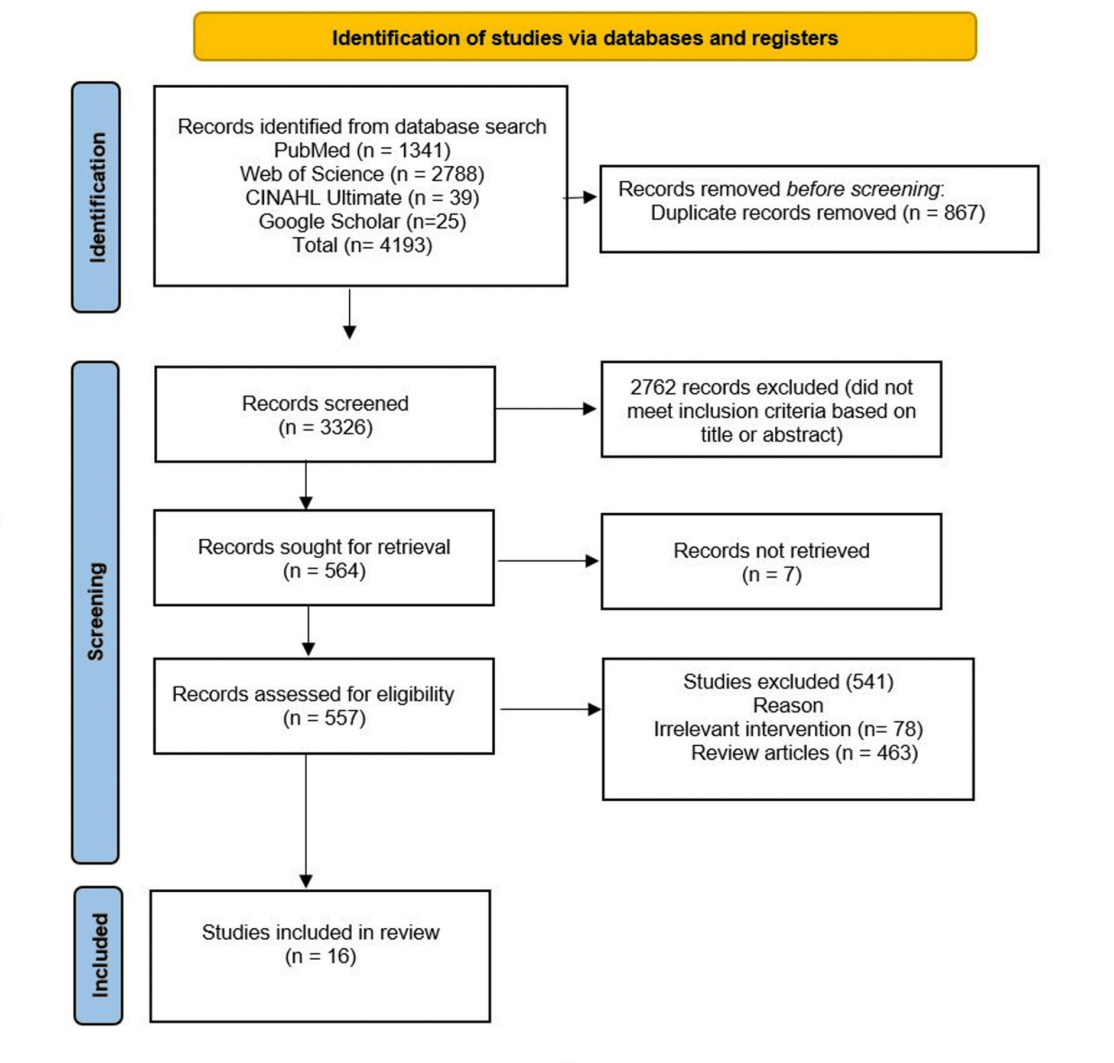
PRISMA flow diagram of the systematic review PRISMA: Preferred Reporting Items for Systematic Reviews and Meta-Analyses

Figure 1 shows the PRISMA flow diagram of this systematic review.

Study Characteristics

A total of 20,339 participants were included in the 16 studies. Regarding study design, 11 were prospective, two were retrospective, two were randomized, and one was a longitudinal study. The age of the included participants ranged from 18 to 47 years. Of the included studies, five studies mainly focused on contraception, whereas 11 mainly targeted conception rates. Digital fertility awareness-based method was the most common method used in four studies, whereas a similar number of studies (n=4) used digital tracking method or cervical mucus monitoring (CMM), or both. The Creighton Model FertilityCare System was used in two studies. App-connected ovulation test system, hormonal contraception (HC) vs. contraceptive mobile application, natural fertility indicators, Marquette model, CMM, and self-observation with the Sensiplan method were used in one study each. The success rate could only be calculated from 13 studies, which showed an average success rate of 69.5%. However, five of these studies reported success rates exceeding 90%. In some studies, the success rate was not explicitly stated. For example, Stanford et al. [16] reported that the probability of conception was increased by 20% with their planning method. Table 1 shows the details of the main findings of the included studies. 

**Table 1:** Demographic details and main findings of the included studies NFP: Nurse-managed natural family planning; EHFM: Electronic hormonal fertility monitor; CMM: Cervical mucus monitoring; TTP: Time to pregnancy

Authors	Year	Type of Study	Participants	Age (years)	Purpose	Method Used	Success Rate	Main Findings
Fehring and Schneider [17]	2017	Prospective study	663	30.4±6.3	Contraceptive	NFP or CMM or both	98%	Unintended pregnancy rates were 2 per 100 women at 24 cycles with correct use and 15 per 100 with typical use. Women using fertility monitor had a lower typical use unintended pregnancy rate (6 per 100) compared to those using cervical mucus monitoring (19 per 100) or both methods combined (18 per 100).
Fehring et al. [18]	2017	Longitudinal study	816	30.3±4.5	Contraceptive	NFP or CMM or both	97%	Correct use pregnancy rates were 3 per 100 users over 12 cycles, while typical use rates were 14 per 100. At 12 cycles, pregnancy rates were 16 per 100 for electronic hormone fertility monitor users, 81 per 100 for mucus-only users, and 14 per 100 for combined monitor plus mucus users.
Pearson et al. [19]	2021	Prospective study	5879	30	Contraceptive	Digital fertility awareness-based method	92.8%	The Natural Cycles app had a one-year typical use Pearl Index (PI) of 6.2 and a perfect use PI of 2.0, with a 13-cycle cumulative pregnancy probability of 7.2%.
Jennings et al. [20]	2019	Prospective study	718	29	Contraceptive	Digital fertility awareness-based method	95%	The perfect-use failure rate of the Dot app was 1.0% (95% CI: 0.9%, 2.9%), while the typical-use failure rate was 5.0% (95% CI: 3.4%, 6.6%) among women aged 18-39.
Favaro et al. [21]	2021	Prospective study	5,376	18-45	To conceive	Digital fertility awareness-based method	61%	The six-cycle and 12-cycle cumulative pregnancy probabilities were 61% (95% CI: 59-62) and 74% (95% CI: 73-76). Women under 35 with regular cycles and frequent intercourse (≥20% of days) had the highest fecundability, achieving an 88% (95% CI: 85-91) pregnancy probability by six cycles and 95% (95% CI: 94-97) by 12 cycles, with a TTP of two cycles.
Stanford et al. [16]	2020	Prospective study	8363	29.9	To conceive	Digital fertility awareness-based method	Probability increased by 20%	Use of any menstrual cycle tracking app was associated with a 12-20% increase in fecundability per cycle, with little difference between selected apps (Clue, Fertility Friend, Glow, Kindara, Ovia) and other apps. Higher fecundability was observed when apps were used alongside fertility indicators (e.g., basal body temperature, cervical fluid).
Johnson et al. [22]	2020	Randomized controlled trial	844	18-40	To conceive	App-Connected Ovulation Test System	36.2%	Women using the ovulation test system had significantly higher pregnancy rates after one cycle (25.4% vs. 14.7%; p < 0.001) and two cycles (36.2% vs. 28.6%; p = 0.026) compared to those not using ovulation testing.
Berglund Scherwitzl et al. [23]	2019	Prospective study	2874	28.1±3.9	To conceive	Hormonal contraception (HC) vs. contraceptive mobile application	2.3 cycles	Women who previously used mobile app had a shorter average time to pregnancy (2.3 cycles) compared to those who discontinued hormonal contraception (3.7 cycles). The time to reach a 30% pregnancy probability was 1.6 times longer for women previously using hormonal contraception, but there was no significant difference in pregnancy probability after 13 cycles.
Bouchard et al. [24]	2017	Prospective study	256	29.2	To conceive	Natural fertility indicators	78%	The overall pregnancy rate was 78 per 100 women over 12 menstrual cycles, with the highest 12-cycle pregnancy rate in the monitor group (83 per 100 women), followed by the mucus group (72) and the combined group (75). Pregnancy rates reached 100% at 24 cycles for women using the hormonal fertility monitor.
Stanford et al. [25]	2021	Retrospective study	370	34.8	To conceive	Creighton Model FertilityCare System	29%	The cumulative live birth rate at two years was 29% overall, with higher rates observed in women under 35 (34%). Of the 63 births with available data, 92% occurred at term, and there were no higher-order multiple gestations, with only two sets of twins.
Mu & Fehring [26]	2014	Prospective study	124	NA	To conceive	Electronic hormonal fertility monitors or CMM or both	87%	Pregnancy rate was 87 per 100 women at 12 months when intercourse occurred on high or peak fertile days, compared to 5 per 100 when intercourse occurred only on low fertile days. Chi-square analysis showed a significantly higher pregnancy rate when intercourse was timed with high or peak fertile days (x2 = 40.2, p < .001).
Stanford et al. [27]	2014	Parallel randomized trial	143	18-35	To conceive	Creighton Model FertilityCare System	36%	The adjusted hazard ratio for the effect of intervention on TTP was 0.86 (95% CI: 0.53, 1.38), indicating no significant impact on TTP. Fecundability in the first cycle was lower for (4%) compared to the control group (17%), with a statistically significant difference (p = 0.02). However, there was no significant difference in fecundability over the entire study period (31% in controls vs. 36% with intervention, p = 0.32).
Marshell et al. [28]	2021	Prospective study	384	33.1±4.5	To conceive	CMM	62.5%	Fertility-awareness instruction using mucus symptom observations helped 62.5% of couples achieve pregnancy, with pregnancy rates 30% higher in the 'high pregnancy-potential' group (72.3% vs 44.4%). Fertile mucus symptoms were associated with a significantly shorter time to conception (4.2 vs 6.4 months).
Frank-Herrmann et al. [29]	2017	Prospective study	187	21-47	To conceive	Self-observation with Sensiplan method	38%	The cumulative pregnancy rate after eight months of fertility awareness training using the Sensiplan method was 38% compared to 21.6% pregnancy rate in untrained couples. Women aged over 35 and couples who had been trying to conceive for more than two years had significantly lower pregnancy rates, with 25% and 17%, respectively.
Fehring & Schneider [30]	2014	Prospective study	197	29.7±5.4	To conceive	EHFM or CMM	13.25 vs 13.68 days, respectively for EHFM and CMM	The EHFM group had fewer days of estimated fertility compared to the CMM group (13.25 vs 13.68 days, respectively; p = .039). The EHFM group had significantly more coital acts than the CMM group (4.22 vs 4.05 acts, respectively; p = .026).
Mu et al. [31]	2022	Retrospective study	1221	_	Contraceptive	Marquette Model NFP	93.3%	The Marquette Model NFP system showed a typical use unintended pregnancy rate of 6.7 per 100 women over 12 months, with lower rates for women with regular cycles (2.8 per 100) and higher rates for postpartum/breastfeeding women (8.0 per 100).

Quality Assessment

**Figure 2:**
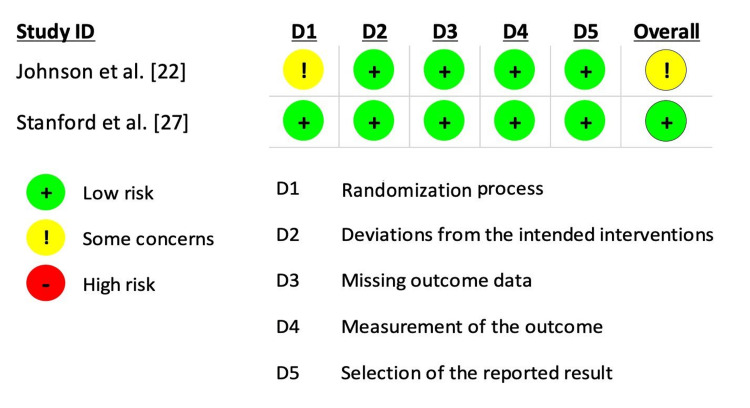
Risk of bias of randomized studies

Only two studies used a randomized controlled study design and were assessed with the ROB 2 tool. Only Johnson et al. [22] reported some concern regarding the randomization process. All other domains including deviation from intended interventions, missing data, outcome assessment, and reported results had low risk (Figure 2).

Table 2 shows the risk of bias performed for retrospective and prospective studies. Only Favaro et al. [21] had a moderate risk of bias, whereas all other studies had low risk of bias. Most studies had risk of bias in the representativeness of the exposed cohorts and controls for other risk factors. Stanford et al. [25] had the highest quality score.

**Table 2:** Risk of bias measured with the Newcastle-Ottawa Scale (NOS)

Study	Selection				Comparability		Outcome			Total quality score
	Representativeness of the exposed cohort	Selection of the non-exposed cohort	Ascertainment of exposure	Demonstration that outcome of interest was not present at the start of study	Controls for the most important risk factors	Controls for other risk factors	Assessment of outcome	Was follow-up long enough for outcomes to occur	Adequacy of follow-up of cohorts	
Fehring and Schneider [17]	1	1	1	1	1	0	1	1	1	8
Fehring et al. [18]	1	1	1	1	1	0	1	1	1	8
Pearson et al. [19]	1	1	1	1	1	0	1	1	1	8
Jennings et al. [20]	1	1	1	1	1	0	0	1	1	7
Favaro et al. [21]	0	1	1	1	1	0	1	0	1	6
Stanford et al. [16]	0	1	1	1	1	0	1	1	1	7
Berglund Scherwitzl et al. [23]	0	1	1	1	1	1	1	1	1	8
Bouchard et al. [24]	1	1	1	1	1	1	1	0	1	8
Stanford et al. [25]	1	1	1	1	1	1	1	1	1	9
Mu & Fehring [26]	0	1	1	1	1	1	1	1	1	8
Marshell et al. [28]	0	1	1	1	1	1	1	1	1	8
Frank-Herrmann et al. [29]	1	1	1	1	1	1	1	0	1	8
Fehring & Schneider [30]	0	1	1	1	1	1	1	1	1	8
Mu et al. [31]	0	1	1	1	1	1	1	1	1	8

Discussion

The analysis of data from 20,339 participants from 16 included studies showed that FABMs can achieve significant success rates when used correctly. The use of FABMs was associated with success in more than half of the cases; however, when used correctly, it resulted in a success rate of over 90%. Our findings align with several previous systematic reviews that have evaluated the efficacy of FABMs on family planning [7,11]. For example, Urrutia et al. in their systematic review reported that first-year typical use pregnancy rates varied widely across fertility awareness-based methods, ranging from as low as 1.8 (Sensiplan and Marquette Monitor methods used for contraception) to as high as 33.6 (Billings Ovulation Method used for both conception and contraception) [5]. Another common factor identified in the present systematic review, with Urrutia et al., was the lack of studies on each method assessing their efficacy. 

Similarly, Duane et al., in their systematic review, reported that FABMs are effective for family planning, and even physicians can also use them effectively to restore reproductive and endocrine systems [7]. Other reviews that have evaluated the efficacy of FABMs have also supported their use for family planning. A systematic review by Grimes et al. included only randomized controlled trials in their analysis [32]. Their findings did not reveal any significant difference among the different FABMs. They also highlighted the paucity of randomized trials, which was also seen in the present systematic review, as only two randomized trials were found. Although randomized trials provide the highest level of evidence, dividing women into different FABMs can be ethically and logistically challenging. Manhart et al., in their review, which included both randomized controlled trials and non-randomized studies, reported that when FABMs were used correctly, they resulted in less than 5% unintended pregnancy rates [4]. However, their systematic review did not clearly describe the evidence base. Compared to the previous systematic review, the current systematic review provides the latest evidence as the search was limited to the last 10 years only. 

The current systematic review also identified that in most studies, women used apps or electronic tracking systems to identify more fertile days. This finding is explainable as the use of technology has increased tremendously over the years. Mobile apps can successfully track fertile days by not only tracking menstruation but also physiological parameters using sophisticated mathematical algorithms [33]. In mobile applications, several parameters, when analyzed together, can lead to higher probability accuracy. For example, in a study by Bull et al., data was analyzed from 612,613 ovulatory cycles, which showed that only 20% of participants had menstrual cycle period of 28 days [34]. The mean duration was 29.3 days. Furthermore, over time, the cycle length decreased by 0.18 days. Differences based on BMI were also observed, with women with BMI>35 having 0.4 days higher length of menstrual cycle compared to BMI of 18.5-25 [34]. These intraindividual variations highlight consideration for these factors in FABMs.

The success of FABMs also depends on behavioral factors. Mu and Fehring observed that intercourse timed during the high or peak fertility phases resulted in an 87% pregnancy rate at 12 months compared to a mere 5% when intercourse was limited to low-fertility days [26]. The transition from traditional paper-based or manual methods to digital fertility tracking applications represents a significant paradigm shift in FABMs. Digital tools harness data analytics, machine learning algorithms, and user-friendly interfaces to provide personalized insights into menstrual cycle patterns [35]. However, traditional methods still hold value, especially in contexts where digital tools may not be accessible or preferred due to cultural or personal reasons. The Creighton Model and Sensiplan, for example, continue to be viable options, particularly when delivered within a structured educational framework [25,29].

The application and success of FABMs also depend on the knowledge level of the health professionals, so they can guide their use. However, the knowledge level of health professionals and medical students varies. For example, a study reported that the calendar technique was the most popular FABM technique among medical students at 84.9% [36]. Similarly, another study that conducted logistic regression analysis applied to the 2013-17 National Survey of Family Growth (NSFG) showed that 3.4% of contraceptive users (176 respondents) used FABMs. FABM users were considerably different from those who used other contraception methods in terms of relationship status, education, parity, health insurance, and religious affiliation. After adjusting for sociodemographic variables, FABM users had lower odds of ever using the pill, higher odds of giving up on it owing to dissatisfaction, and higher odds of intending to have more children than users of other contraceptive methods [10]. Another study showed that approximately 18% of contraception users used FABMs [37].

Strengths and limitations

The main strength of this systematic review was that latest evidence was included in the systematic review. Another strength of this systematic review was that systemic search was conducted in various key databases. Furthermore, the systematic review included more than 20,000 participants. Furthermore, both randomized and longitudinal studies were included. The included studies also focused on both contraception and participants trying to conceive. However, there are some limitations as well which should be considered while interpreting the findings. Firstly, most of the studies relied on self-reported data, which could have introduced bias in findings. Another limitation was that studies only included participants who were willing to participate and had higher knowledge levels compared to the general population. This may limit the generalizability of the findings in the general population. 

## Conclusions

The findings of the present study showed that FABMs are effective in family planning, whether the contraception is targeted or the goal is to conceive. Furthermore, the success rate was associated with correct use. Studies showed that when FABMs were used correctly, they were associated with a more than 90% success rate for both conception and contraception purposes. The success rates also varied based on the different FABMs used. Digital methods, in particular, show promise due to their ability to reduce user error through automated tracking and personalized feedback. However, the effectiveness of these methods is heavily contingent upon user education, adherence, and the accurate interpretation of fertility signals. There are some limitations of the included studies as well, which should be addressed in larger studies with a longer follow-up duration.

## Appendices

**Table 3:** Combinations of keywords used in the search strategy and number of resources found

Key variable	Sub terms	Search options	PubMed	Web of Science	CINAHL Ultimate
1. Fertility awareness methods	#1 Fertility awareness	TI/AB	457	1675	275
	#2 Natural family planning	TI/AB	811	1821	183
	#3 Rhythm method	TI/AB	372	31930	376
	#4 Calendar method	TI/AB	107	10626	166
	#5 Cervical mucus method	TI/AB	127	571	12
	#6 Basal body temperature	TI/AB	861	2756	91
	#7 Symptothermal method	TI/AB	50	44	14
	#8 Menstrual tracking apps	TI/AB	11	73	17
	#9 Fertility apps	TI/AB	15	100	34
	#10 Marquette method	TI/AB	9	118	6
(((((((((Fertility awareness[Title/Abstract]) OR (Natural family planning[Title/Abstract])) OR (Rhythm method[Title/Abstract])) OR (Calendar method[Title/Abstract])) OR (Cervical mucus method[Title/Abstract])) OR (Basal body temperature[Title/Abstract])) OR (Symptothermal method[Title/Abstract])) OR (Menstrual tracking apps[Title/Abstract])) OR (Fertility apps[Title/Abstract])) OR (Marquette method[Title/Abstract])			2236	49284	1096
2. Family planning outcomes	#1 Contraception	TI/AB	46080	27404	12040
	#2 Birth control	TI/AB	5874	81178	4095
	#3 Family planning	TI/AB	45702	55318	8936
	#4 Pregnancy prevention	TI/AB	1722	18560	2403
	#5 Contraceptive effectiveness	TI/AB	1584	2213	358
	#6 Unintended pregnancy	TI/AB	3844	5344	3262
(((((Contraception[Title/Abstract]) OR (Birth control[Title/Abstract])) OR (Family planning[Title/Abstract])) OR (Pregnancy prevention[Title/Abstract])) OR (Contraceptive effectiveness[Title/Abstract])) OR (Unintended pregnancy[Title/Abstract])			75053	176645	26195
((((((((((Fertility awareness[Title/Abstract]) OR (Natural family planning[Title/Abstract])) OR (Rhythm method[Title/Abstract])) OR (Calendar method[Title/Abstract])) OR (Cervical mucus method[Title/Abstract])) OR (Basal body temperature[Title/Abstract])) OR (Symptothermal method[Title/Abstract])) OR (Menstrual tracking apps[Title/Abstract])) OR (Fertility apps[Title/Abstract])) OR (Marquette method[Title/Abstract])) AND ((((((Contraception[Title/Abstract]) OR (Birth control[Title/Abstract])) OR (Family planning[Title/Abstract])) OR (Pregnancy prevention[Title/Abstract])) OR (Contraceptive effectiveness[Title/Abstract])) OR (Unintended pregnancy[Title/Abstract]))			1341	2788	39

## References

[1] Fauser BC, Adamson GD, Boivin J (2024). Declining global fertility rates and the implications for family planning and family building: an IFFS consensus document based on a narrative review of the literature. Hum Reprod Update.

[2] Simmons RG, Jennings V (2020). Fertility awareness-based methods of family planning. Best Pract Res Clin Obstet Gynaecol.

[3] Chowdary GD, Choudri VS, Reddy NP (2024). Cross‐sectional study of fertility awareness and reproductive health knowledge among young adults. Res J Med Sci.

[4] Manhart MD, Duane M, Lind A, Sinai I, Golden-Tevald J (2013). Fertility awareness-based methods of family planning: a review of effectiveness for avoiding pregnancy using SORT. Osteopath Fam Physician.

[5] Urrutia RP, Polis CB (2019). Fertility awareness based methods for pregnancy prevention. BMJ.

[6] Ashoor R, Alrashid S, Alruhaimi S, Alanazi S, Alzahrani H, Alshammari YS, Alotaibi A (2023). Awareness of contraceptives and their use among Saudi women attending primary care centers in King Abdulaziz Medical City, Riyadh, Saudi Arabia. Cureus.

[7] Duane M, Stanford JB, Porucznik CA, Vigil P (2022). Fertility awareness-based methods for women's health and family planning. Front Med (Lausanne).

[8] Hampton KD, Newton JM, Parker R, Mazza D (2016). A qualitative study of the barriers and enablers to fertility-awareness education in general practice. J Adv Nurs.

[9] Bellizzi S, Pichierri G, Menchini L, Barry J, Sotgiu G, Bassat Q (2019). The impact of underuse of modern methods of contraception among adolescents with unintended pregnancies in 12 low- and middle-income countries. J Glob Health.

[10] Brewer M, Stevens L (2021). Use of fertility awareness-based methods of contraception: evidence from the National Survey of Family Growth, 2013-2017. Contraception.

[11] Peragallo Urrutia R, Polis CB, Jensen ET, Greene ME, Kennedy E, Stanford JB (2018). Effectiveness of fertility awareness-based methods for pregnancy prevention: a systematic review. Obstet Gynecol.

[12] Moher D, Shamseer L, Clarke M (2015). Preferred reporting items for systematic review and meta-analysis protocols (PRISMA-P) 2015 statement. Syst Rev.

[13] Ouzzani M, Hammady H, Fedorowicz Z, Elmagarmid A (2016). Rayyan-a web and mobile app for systematic reviews. Syst Rev.

[14] Wells G, Shea B, O'Connell D, Peterson J, Welch V, Losos M, Tugwell P (2000). The Newcastle-Ottawa scale (NOS) for Assessing the Quality of Non-randomized Studies in Meta-analysis. https://www.ohri.ca/programs/clinical_epidemiology/oxford.asp.

[15] Sterne JA, Savović J, Page MJ (2019). RoB 2: a revised tool for assessing risk of bias in randomised trials. BMJ.

[16] Stanford JB, Willis SK, Hatch EE, Rothman KJ, Wise LA (2020). Fecundability in relation to use of mobile computing apps to track the menstrual cycle. Hum Reprod.

[17] Fehring RJ, Schneider M (2017). Effectiveness of a natural family planning service program. MCN Am J Matern Child Nurs.

[18] Fehring RJ, Schneider M, Bouchard T (2017). Effectiveness of an online natural family planning program for breastfeeding women. J Obstet Gynecol Neonatal Nurs.

[19] Pearson JT, Chelstowska M, Rowland SP (2021). Contraceptive effectiveness of an FDA-cleared birth control app: results from the natural cycles U.S. cohort. J Womens Health (Larchmt).

[20] Jennings V, Haile LT, Simmons RG, Spieler J, Shattuck D (2019). Perfect- and typical-use effectiveness of the Dot fertility app over 13 cycles: results from a prospective contraceptive effectiveness trial. Eur J Contracept Reprod Health Care.

[21] Favaro C, Pearson JT, Rowland SP (2021). Time to pregnancy for women using a fertility awareness based mobile application to plan a pregnancy. J Womens Health (Larchmt).

[22] Johnson S, Stanford JB, Warren G, Bond S, Bench-Capon S, Zinaman MJ (2020). Increased likelihood of pregnancy using an app-connected ovulation test system: a randomized controlled trial. J Womens Health (Larchmt).

[23] Berglund Scherwitzl E, Lundberg O, Kopp Kallner H (2019). Short- and long-term effect of contraceptive methods on fecundity. Eur J Contracept Reprod Health Care.

[24] Bouchard TP, Fehring RJ, Schneider MM (2017). Achieving pregnancy using primary care interventions to identify the fertile window. Front Med (Lausanne).

[25] Stanford JB, Carpentier PA, Meier BL, Rollo M, Tingey B (2021). Restorative reproductive medicine for infertility in two family medicine clinics in New England, an observational study. BMC Pregnancy Childbirth.

[26] Mu Q, Fehring RJ (2014). Efficacy of achieving pregnancy with fertility-focused intercourse. MCN Am J Matern Child Nurs.

[27] Stanford JB, Smith KR, Varner MW (2014). Impact of instruction in the Creighton model fertilitycare system on time to pregnancy in couples of proven fecundity: results of a randomised trial. Paediatr Perinat Epidemiol.

[28] Marshell M, Corkill M, Whitty M, Thomas A, Turner J (2021). Stratification of fertility potential according to cervical mucus symptoms: achieving pregnancy in fertile and infertile couples. Hum Fertil (Camb).

[29] Frank-Herrmann P, Jacobs C, Jenetzky E (2017). Natural conception rates in subfertile couples following fertility awareness training. Arch Gynecol Obstet.

[30] Fehring RJ, Schneider M (2014). Comparison of abstinence and coital frequency between 2 natural methods of family planning. J Midwifery Womens Health.

[31] Mu Q, Fehring RJ, Bouchard T (2022). Multisite effectiveness study of the Marquette method of natural family planning program. Linacre Q.

[32] Grimes DA, Gallo MF, Grigorieva V, Nanda K, Schulz KF (2004). Fertility awareness-based methods for contraception. Cochrane Database Syst Rev.

[33] Johnson S, Marriott L, Zinaman M (2018). Can apps and calendar methods predict ovulation with accuracy?. Curr Med Res Opin.

[34] Bull JR, Rowland SP, Scherwitzl EB, Scherwitzl R, Danielsson KG, Harper J (2019). Real-world menstrual cycle characteristics of more than 600,000 menstrual cycles. NPJ Digit Med.

[35] Schantz JS, Fernandez CS, Anne Marie ZJ (2021). Menstrual cycle tracking applications and the potential for epidemiological research: a comprehensive review of the literature. Curr Epidemiol Rep.

[36] Alenezi GG, Haridi HK (2021). Awareness and use of family planning methods among women in Northern Saudi Arabia. Middle East Fertil Soc J.

[37] Polis CB, Otupiri E, Bell SO, Larsen-Reindorf R (2021). Use of fertility awareness-based methods for pregnancy prevention among Ghanaian women: a nationally representative cross-sectional survey. Glob Health Sci Pract.

